# CORRELATION BETWEEN FRAILTY AND DNA DAMAGE IN HEMATOPOIETIC STEM CELLS: A PILOT STUDY

**DOI:** 10.1093/geroni/igz038.333

**Published:** 2019-11-08

**Authors:** Paolo Mazzola, Silvia Bombelli, Chiara Grasselli, Maddalena Bolognesi, Laura Antolini, Giorgio Cattoretti, Giorgio Annoni, Roberto Perego

**Affiliations:** 1 University of Milano-bicocca, School of medicine and surgery, Monza, Italy; 2 University of Milano-Bicocca, Department of Medicine and Surgery, Monza, Italy

## Abstract

Frailty is an age-related syndrome characterized by a progressive impairment of multiple physiological systems and leading to poor clinical and functional outcomes. Our aim was to explore the DNA damage, as an effect of increased oxidative stress related to frailty, on peripheral blood mononuclear cells (PBMC) and circulating hematopoietic stem cells (cHSC). According to Fried’s operating definition of frailty, we enrolled three groups of subjects: frail seniors (age >65 years, n=19), fit seniors (>65 years, n= 16) and young controls (age 25-35 years, n=19). We carried out a comprehensive assessment and obtained 3 vials of whole blood for cells and plasma separation. We separated PBMC by Ficoll-Paque and stained with specific conjugated antibodies leucocyte lineage and HSC. We evaluated DNA damage by FACS detection of γ-H2AX in the total PBMC and cHSC subpopulation. We observed an increased percentage of cells, although not significant, with DNA damage in PBMC from frail seniors (0.70%) compared to fit seniors (0.37%) and young controls (0.13%). The percentage of cells with DNA damage in cHSC of frail seniors (2.97%) was higher compared to fit seniors (1.66%, not significant) and young controls (0.46%, statistically significant). Moreover, cHSC present the statistically higher amount of DNA damage, measured as fluorescence intensity, compared to fit seniors and young controls. cHSC from frail seniors show the highest total DNA damage, compared to fit seniors and young controls. This is probably linked to an increase of oxidative stress related to frailty, which we are going to analyze in the near future.

